# Effect of short-term mindfulness-based stress reduction on sleep quality in male patients with alcohol use disorder

**DOI:** 10.3389/fpsyt.2023.928940

**Published:** 2023-03-14

**Authors:** Yongmei Wang, Cuiping Chen, Lina Gu, Yi Zhai, Yanhong Sun, Guoqing Gao, Yayun Xu, Liangjun Pang, Lianyin Xu

**Affiliations:** ^1^Department of Nursing, Affiliated Psychological Hospital of Anhui Medical University, Hefei, China; ^2^Anhui Mental Health Center, Hefei, China; ^3^Department of Nursing, Hefei Fourth People's Hospital, Hefei, China; ^4^Department of Material Dependence, Affiliated Psychological Hospital of Anhui Medical University, Hefei, China; ^5^Department of Material Dependence, Hefei Fourth People's Hospital, Hefei, China; ^6^Department of Pharmacy, Affiliated Psychological Hospital of Anhui Medical University, Hefei, China; ^7^Department of Pharmacy, Hefei Fourth People's Hospital, Hefei, China; ^8^Department of Epidemiology and Biostatistics, School of Public Health, Anhui Medical University, Hefei, China

**Keywords:** cardiopulmonary coupling, sleep quality, apnea index, alcohol use disorder, mindfulness-based stress reduction

## Abstract

**Background:**

Sleep disturbance is one of the most prominent complaints of patients with alcohol use disorder (AUD), with more than 70% of patients with AUD reporting an inability to resolve sleep problems during abstinence. Mindfulness-based stress reduction (MBSR) has been shown to improve sleep quality and as an alternative therapy to hypnotics for sleep disorders.

**Objective:**

The aim of the present study was to evaluate the effect of short-term MBSR on sleep quality in male patients with AUD after withdrawal.

**Methods:**

A total of 91 male patients with AUD after 2 weeks of routine withdrawal therapy were randomly divided into two groups using a coin toss: the treatment group (*n* = 50) and the control group (*n* = 41). The control group was received supportive therapy, and the intervention group added with MBSR for 2 weeks on the basis of supportive therapy. Objective sleep quality was measured at baseline and 2 weeks after treatment using the cardiopulmonary coupling (CPC). Indicators related to sleep quality include total sleep time, stable sleep time, unstable sleep time, rapid eye movement (REM) sleep time, wake-up time, stable sleep latency, sleep efficiency, and apnea index. These indicators were compared by an analysis of covariance (ANCOVA) between the two groups, controlling for individual differences in the respective measures at baseline.

**Results:**

The results showed that there were no significant differences in the age [*t* (89) = –0.541, *P* = 0.590), BMI [*t* (89) = –0.925, *P* = 0.357], educational status [*t* (89) = 1.802, *P* = 0.076], years of drinking [*t* (89) = –0.472, *P* = 0.638), daily intake [*t* (89) = 0.892, *P* = 0.376], types of alcohol [χ^2^ (1) = 0.071, *P* = 0.789], scores of CIWA-AR [*t* (89) = 0.595, *P* = 0.554], scores of SDS [*t* (89) = –1.151, *P* = 0.253), or scores of SAS [*t* (89) = –1.209, *P* = 0.230] between the two groups. Moreover, compared with the control group, the total sleep time [*F* (1.88) = 4.788, *P* = 0.031) and stable sleep time [*F* (1.88) = 6.975, *P* = 0.010] were significantly increased in the treatment group. Furthermore, the average apnea index in the patients who received MBSR was significantly decreased than in the control group [*F* (1.88) = 5.284, *P* = 0.024].

**Conclusion:**

These results suggest that short-term MBSR could improve sleep quality and may serve as an alternative treatment to hypnotics for sleep disturbance in patients with AUD after withdrawal.

## 1. Introduction

According to the 2018 World Health Organization Global status report on alcohol and health, the harmful use of alcohol is responsible for ~3 million deaths, accounting for nearly 5.3% of the global deaths due to disease in 2016 (1). Beyond health problems, the harmful use of alcohol imposes high social and economic costs on society (2). Due to the fact that approximately 90% of patients with alcohol use disorder (AUD) experience at least one relapse over a 4-year period after treatment, reducing the craving and recurrence rate is of great clinical significance (3).

Sleep disturbance is one of the most prominent complaints of alcohol-dependent patients, with more than 70% of patients with AUD reporting an inability to resolve sleep problems during abstinence (4). Accumulated data support a bidirectional relationship between alcohol craving and sleep disturbance (5–9). It has been reported that alcohol negatively affects sleep quality by decreasing the rapid eye movement (REM) sleep phase and the total sleep time (10). On the contrary, patients with obstructive sleep apnea are at a higher risk of developing alcohol-related diseases (8). Most alcohol-dependent patients may use alcohol to self-medicate sleep disturbance (11). Given that sleep disturbance is associated with negative consequences such as decreased quality of life, poor work performance, and mood and anxiety symptoms, improving sleep quality in alcohol recovery may provide an easily identifiable treatment target and potentially improve long-term outcomes of alcohol dependence. However, treatment options for sleep disorders in patients with AUD are limited, and there may be risks due to abuse/overdose associated with some hypnotics (12).

Mindfulness meditation was developed to help individuals manage stress or illnesses by combining meditation with mindfulness skills training (13). Growing evidence supports the benefits of meditation for ameliorating sleep quality (14–17). Mindfulness-based stress reduction (MBSR), a standardized stress reduction program that emphasizes mindfulness meditation, has recently been shown to significantly improve sleep disturbance symptoms in several diseases, including cancers, cirrhosis, and insomnia (18–21). However, to our knowledge, few studies have evaluated the potential therapeutic effect of MBSR on sleep disturbances in patients with AUD. Given the good safety and potential therapeutic effects on sleep quality, MBSR may be a useful, non-pharmacological, and non-invasive method with a beneficial impact on the sleep management of patients with AUD.

Considering the close relationship between sleep disturbance and negative consequences of AUD (22, 23), together with the potential therapeutic effect of MBSR on sleep quality, the aim of the present study was to investigate the effect of short-term MBSR on sleep quality in male patients with AUD after withdrawal.

## 2. Materials and methods

### 2.1. Subjects

The study was conducted between October 2019 and October 2020 at the Affiliated Psychological Hospital of Anhui Medical University. A total of 91 male patients with a diagnosis of AUD were included as per the International Classification of Diseases 10th Revision Diagnostic Criteria for Research (ICD-10 DCR) criteria. All the patient subjects conformed to the following inclusion criteria: (1) male inpatients; (2) age 18–65 years; (3) Han Chinese ethnicity; (4) completion of benzodiazepines substitution. Patients with any of the following were excluded from this study: (1) any comorbid psychiatric disorders (e.g., psychosis, schizophrenia, bipolar disorder, panic disorder, and obsessive–compulsive disorder) or active suicidal ideation (moderate or high suicidality); (2) diagnosed with a substance-dependent disorder other than alcohol; and (3) diagnosed with a serious neurological or medical condition. In accordance with the principles of the Declaration of Helsinki, all subjects provided informed written consent prior to participation. The Ethics Committee of Hefei Fourth People's Hospital, Anhui Mental Health Center, approved this study.

### 2.2. Demographic and clinical data collection

A Demographics Questionnaire was used to collect general information about participants, such as sex, age, body weight, height, body mass index (BMI), years of education, marital status, years of drinking, daily intake, and types of alcohol by face-to-face interview. The severity of alcohol withdrawal was measured by the Clinical Institute Withdrawal Assessment-Alcohol, Revised (CIWA-AR), which is a 10-item measure used to provide a quantitative index of the severity of the alcohol withdrawal syndrome. The most common mental health comorbidities associated with AUD were anxiety and depression disorder (24). The Self-Rating Anxiety Scale (SAS) and the Self-Rating Depression Scale (SDS) were used to assess the degree of anxiety and depression, respectively.

### 2.3. Study design

**Figure 1:**
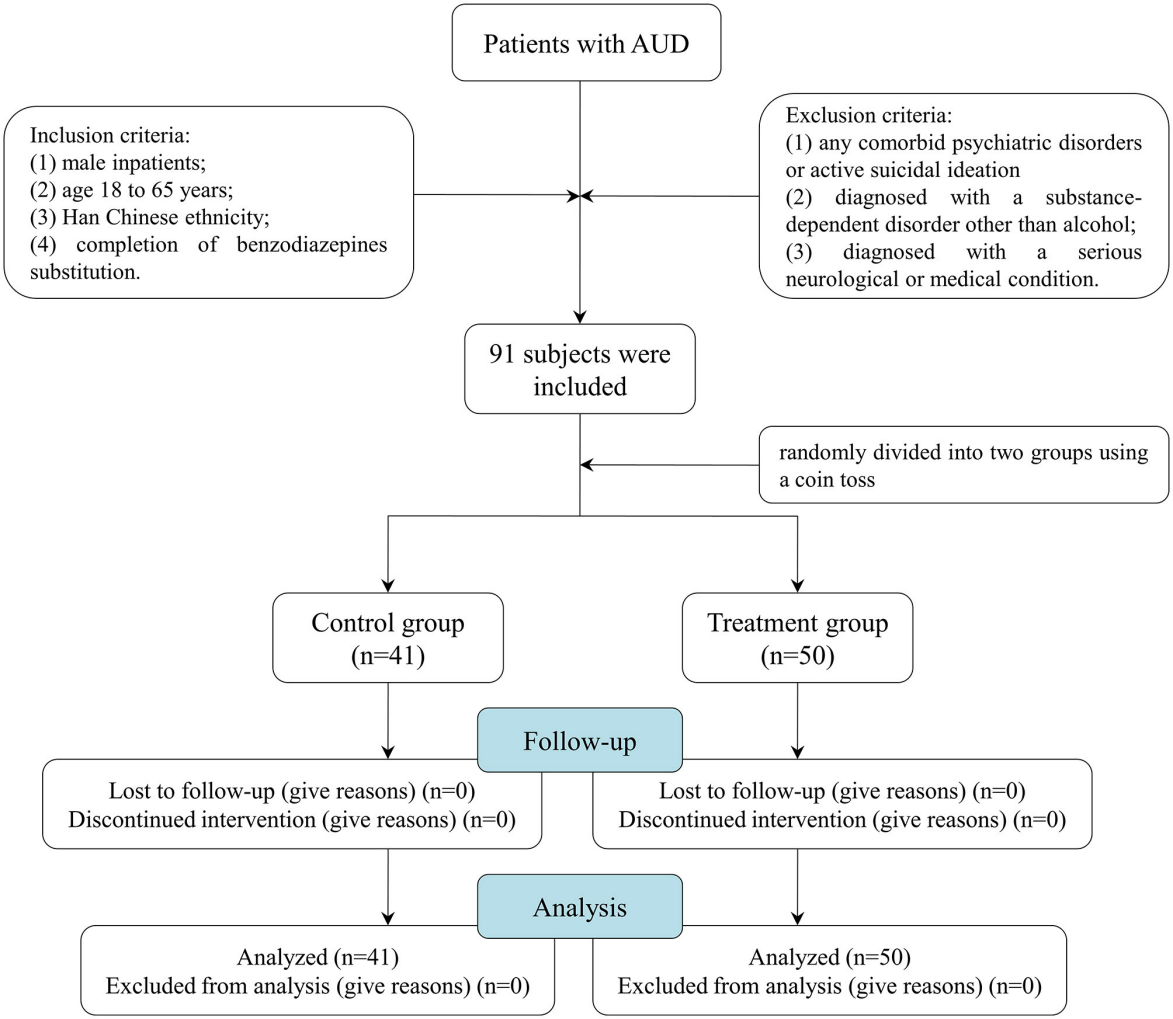
Flowchart showing the enrollment process for participants.

A total of 91 male patients with AUD were enrolled. These patients all underwent an alcohol dependence rehabilitation treatment program for 1 month, including 1-week detoxification and 3-week supportive therapy, such as correction of electrolyte imbalance, liver-protecting therapy, and vitamin B supplementation. All patients should meet the conditions of receiving detoxification replacement therapy to reduce dependence (1 week) and be in a stable alcohol withdrawal period at the time of enrollment before MBSR treatment. At the end of the 2nd week of the treatment program, they were randomly divided into two groups using a coin toss: the treatment group (*n* = 51) and the control group (*n* = 40). The control group received supportive therapy, and the treatment group was provided with MBSR for 2 weeks on the basis of supportive therapy. The training was conducted once a day for 45 min, 5 times/week, for 2 weeks. Before the first intervention, the trained nurses introduced the meditation in detail, including the theoretical knowledge, efficacy, and precautions of meditation training, to ensure that each patient was clear and mastered the training procedure. The patients were placed in a sound insulation room with a constant temperature of 25°C and asked to sit upright with their backs against the backrest of an armless chair. The procedures of meditation were as follows: The trained nurses used simple instructions to lead the patients to experience a meditative state of concentration for 10 min and then played a series of meditation music to guide the patients to conduct a 35-min meditation relaxation training: (1) focus on breathing and make it deep and long; (2) completely relax the muscles of the whole body from head to feet; (3) feel any subtle changes in mind, and experience the changes of the body from feet to head; (4) imagine pleasant things or scenes to completely relax body and mind; and (5) slowly open eyes and end the meditation (for a more detailed script of the MBSR, see Supplementary Table S1). Objective sleep quality was measured at baseline and 2 weeks after treatment using the cardiopulmonary coupling (CPC; Fengshengyongkang Software Technology Ltd., Co., Nanjing, Jiangsu Province, China). Figure 1 illustrates the summary of the participants' screening and enrollment processes.

### 2.4. CPC analysis

The CPC technique is based on a continuous single-lead electrocardiogram (ECG) signal and uses the Fourier transform to extract heart rate variability and ECG-derived respiration (EDR) activity from the ECG signal. Sleep stability and the presence of sleep-disordered breathing can be obtained from the connected software by calculating the cross-power and coherence between the two signals. Specifically, high-frequency coupling (frequency range of 0.1–0.4 Hz) represents stable sleep; low-frequency coupling (frequency range of 0.01–0.1 Hz) represents unstable sleep; very-low-frequency coupling (frequency range of 0.001–0.01 Hz) represents wakefulness or rapid eye movement sleep; elevated-low frequency coupling represents apnea index [specifically, the detection of elevated-low-frequency coupling requires that the minimum low-frequency power be >0.05 normalized units and that the low to high-frequency ratio be >30 to define periods of probable apnea/hypopnea (25). The apnea index defines as the average number of apneas and hypopneas occurrences per hour].

### 2.5. Statistical analysis

The data were analyzed using SPSS (Version 17.0; SPSS, Inc, Chicago, IL, USA). The differences in the demographic and clinical characteristics were analyzed using Student's *t*-test or χ^2^ test between groups. After a 2-week treatment, an analysis of covariance (ANCOVA) was performed to compare the indicators related to sleep quality, including total sleep time, stable sleep time, unstable sleep time, REM sleep time, wake-up time, stable sleep latency, sleep efficiency, and apnea index between groups, controlling for individual differences on the respective measures at baseline. An interaction term between the group and the covariate was initially included to check the parallelism assumption, and all the interactions were not significant. A *P*-value of < 0.05 (two-tailed) was considered statistically significant.

A sensitivity analysis using G-power software (Version 3.1.9.2 Heinrich-Heine-University Düsseldorf, Düsseldorf, Germany) (26) indicated that, given α of 0.05 and power (1-β) of 0.80, a study with two groups and a total sample size of 91 would be able to detect an effect size (f) of 0.30 with fixed effects, main effects and interactions ANCOVA (noncentrality parameter λ = 8.02; critical F = 3.95; denominator df = 88). Using Cohen's (27) effect size guidelines of *f* = 0.10 as small, *f* = 0.25 as medium, and *f* = 0.40 as large, our sample size was sufficient to detect medium effect sizes. Therefore, the study is sufficiently powered to detect moderate differences in the CPC-based sleep quality between groups.

## 3. Results

### 3.1. Demographic and clinical characteristics of the treatment and control groups

As shown in Table 1, there were no significant differences in the age (*t* (89) = −0.541, *P* = 0.590), BMI (*t* (89) = −0.925, *P* = 0.357), educational status (*t* (89) = 1.802, *P* = 0.076), marital status (χ^2^ (1) = 1.745, *P* = 0.186), years of drinking (*t* (89) = −0.472, *P* = 0.638), daily intake (*t* (89) = 0.892, *P* = 0.376), types of alcohol (χ^2^ (1) = 0.071, *P* = 0.789), scores of CIWA-AR (*t* (89) = 0.595, *P* = 0.554), scores of SDS (*t* (89) = −1.151, *P* = 0.253), or scores of SAS (*t* (89) = −1.209, *P* = 0.230) between the two groups.

**Table 1:** Comparison of demographic and clinical data in the treatment and control groups (mean ± SEM).

**Variables**	**Control group**	**Treatment group**	**Statistics (*t*/χ^2^)**	***P***
Age	44.10 ± 1.48	45.18 ± 1.35	−0.541	0.590
BMI (kg/m^2^)	21.80 ± 0.56	22.47 ± 0.47	−0.925	0.357
Educational status (years)	10.07 ± 0.53	8.88 ± 0.39	1.802	0.076
Marital status			1.745	0.186
Married	34	46		
Single/divorced/separated	7	4		
Years of drinking	20.16 ± 1.66	21.18 ± 1.41	−0.472	0.638
Daily intake (standard drink)	9.51 ± 0.52	8.74 ± 0.72	0.892	0.376
Types of alcohol			0.071	0.789
Wine	36	45		
Wine + Beer	4	6		
CIWA-AR	6.67 ± 0.73	6.03 ± 0.76	0.595	0.554
SDS	42.03 ± 1.60	44.85 ± 1.86	−1.151	0.253
SAS	38.16 ± 1.56	40.80 ± 1.51	−1.209	0.230

### 3.2. Effects of short-term MBSR on the CPC-based sleep quality in patients with AUD

**Figure 2:**
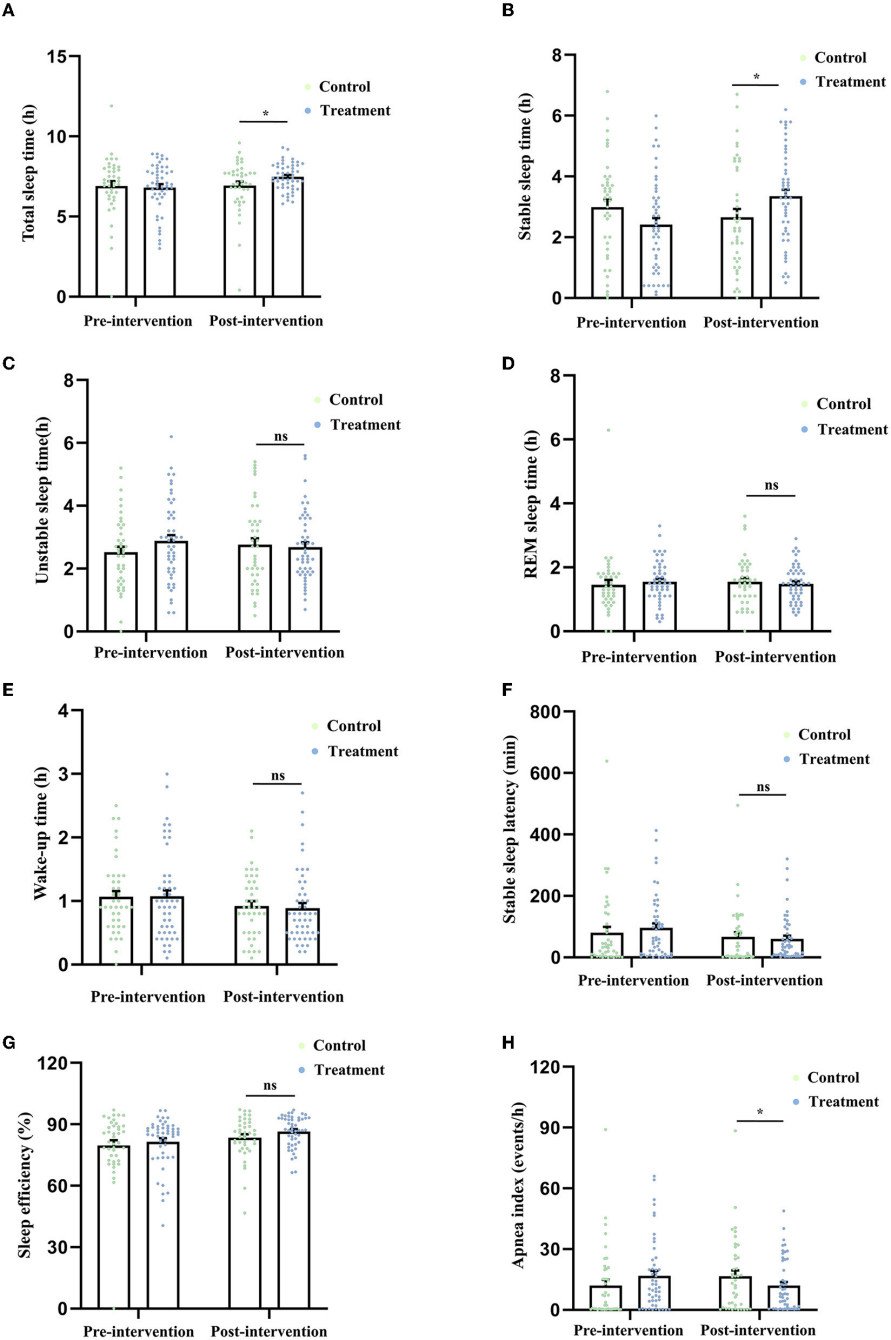
Comparison of the indicators related to sleep quality derived from the CPC technique, including total sleep time **(A)**, stable sleep time **(B)**, unstable sleep time **(C)**, REM sleep time **(D)**, wake-up time **(E)**, stable sleep latency **(F)**, sleep efficiency **(G)**, and apnea index **(H)** between the two groups after 2-week treatment. *P < 0.05 was considered statistically significant. ns, no significance.

The total sleep time of the control group and the treatment group at baseline was (6.91 ± 0.29) h and (6.81 ± 0.22) h, respectively. After 2-week treatment, the results of the ANCOVA showed that compared with the control group, the total sleep time in the treatment group was significantly higher (*F* (1.88) = 4.788, *P* = 0.031, Figure 2A).

The stable sleep time of the control group and the treatment group at baseline was (2.98 ± 0.26) h and (2.41 ± 0.22) h, respectively. After the treatment, the stable sleep time in the patients who received MBSR was significantly higher than that in the control group [*F* (1.88) = 6.975, *P* = 0.010, Figure 2B].

The unstable sleep time of the control group and the treatment group at baseline was (2.51 ± 0.18) h and (2.88 ± 0.18) h, respectively. After the treatment, there was no difference in unstable sleep time [*F* (1.88) = 1.241, *P* = 0.268, Figure 2C] between the two groups.

The REM sleep time of the control group and the treatment group at baseline was (1.45 ± 0.15) h and (1.54 ± 0.09) h, respectively. After the treatment, there was no difference in the REM sleep time [*F* (1.88) = 0.810, *P* = 0.371, Figure 2D] between the two groups.

The wake-up time of the control group and the treatment group at baseline was (1.06 ± 0.09) h and (1.07 ± 0.10) h, respectively. After the treatment, no difference in the wake-up time was found between the two groups [*F* (1.88) = 0.105, *P* = 0.747, Figure 2E].

The stable sleep latency of the control group and the treatment group at baseline was (79.85 ± 19.17) min and (95.82 ± 14.67) min, respectively. After the treatment, no difference in the wake-up time was found between the two groups [*F* (1.88) = 0.184, *P* = 0.669, Figure 2F].

The sleep efficiency (ratio of total sleep time to total in-bed time) of the control group and the treatment group at baseline was (79.75 ± 2.48)% and (81.55 ± 1.70)%, respectively. After the treatment, no difference in the wake-up time was found between the two groups [*F* (1.88) = 2.387, *P* = 0.126, Figure 2G].

The apnea index (average number of apnea occurrences per hour) of the control group and the treatment group at baseline was (12.08 ± 2.76) events/h and (16.74 ± 2.48) events/h, respectively. After the treatment, the apnea index in the treatment group was significantly lower than that in the control group [*F* (1.88) = 5.284, *P* = 0.024, Figure 2H].

## 4. Discussion

Accumulating evidence indicates that MBSR has the potential to improve sleep quality cancers, cirrhosis, and insomnia (18–21, 28). To our knowledge, this is the first study to analyze the effects of MBSR on sleep quality in patients with AUD. The main finding is that 2-week MBSR increased the total sleep time and stable sleep time and decreased the apnea index in patients with AUD, suggesting the potential therapeutic effect of short-term MBSR on sleep disturbance in patients with AUD after withdrawal.

Cardiopulmonary coupling is a spectroscopic method developed as an alternative to polysomnography to quantify sleep quality (29). The CPC technique is based on a continuous single-lead electrocardiogram (ECG) signal to track changes in cardiac inter-beat (R-R) intervals and QRS amplitude during sleep (25). Since CPC analysis has several advantages, such as objectivity, repeatability, automation, and scorer-independence (30), it has been widely used to evaluate sleep quality in several diseases, including Parkinson's disease (31), depression (32), and obstructive sleep apnea (33). Therefore, CPC was chosen to measure sleep quality in the present study.

Several lines of evidence have suggested that MBSR is proposed as an adjunctive treatment targeting relapse prevention in substance use disorders (34), the mechanisms of which may be related to improved emotion regulation, reduced stress reactivity, and decreased risk of relapse in high-risk situations (35, 36). In terms of AUD, several studies of mindfulness-based interventions have shown beneficial findings for risks of relapse and the severity of depression, anxiety, and stress symptoms (37–39). However, to the best of our knowledge, few studies examine the effects of MBSR on sleep quality in patients with AUD. The results of the present study indicated that short-term MBSR as an adjunct to usual care showed to improve sleep disturbance in alcohol-dependent patients in early recovery compared to usual care alone, providing a novel potential mechanism for MBSR as an adjunctive therapy for patients with AUD.

Sleep continuity includes total sleep time, stable sleep time, sleep latency, sleep efficiency, and wake after sleep onset. Among them, stable sleep is characterized by the absence of respiratory abnormality or progressive flow limitation and usually demonstrates non-CAP EEG (40). Numerous studies have confirmed disturbances of sleep continuity in patients with AUD (41–43). Specifically, patients with AUD showed difficulty in falling asleep, decreased total sleep time, and decreased sleep efficiency after acute abstinence, predicting the likelihood of relapse during longer periods of abstinence (44). In the present study, 2-week MBSR was found to increase the total sleep time and stable sleep time in alcohol-dependent patients after withdrawal, suggesting that short-term MBSR could improve the disturbances of sleep continuity in patients with AUD.

Sleep apnea, characterized by recurrent upper airway obstruction during sleep, is associated with arterial blood desaturation, sympathetic nervous system activation, and cardiovascular impairment (45, 46). The apnea index is an indicator of the severity of sleep apnea, which measures the hourly occurrence rate of apneas and hypopneas (47). Previous studies have indicated that alcohol dependence is associated with sleep apnea. Alcohol has been shown to relax the upper airway while reducing the normal arousal response to airway obstruction, leading to impaired normal breathing, such as sleep fragmentation and sleep-disordered breathing (10, 48). A recent study has demonstrated a significant independent association of dispositional mindfulness with continuous positive airway pressure (CPAP) (49), a standard treatment for obstructive sleep apnea. Taken together, the results of the present study show that the apnea index was significantly decreased in patients with AUD who received MBSR compared with control subjects; it is rational to presume that MBSR may be a potential treatment option for sleep apnea in male patients with AUD after withdrawal.

The following medications are currently Food and Drug Administration (FDA) approved for the treatment of sleep disorders: benzodiazepines and non-benzodiazepine hypnotics, tricyclic antidepressants, therapeutic drugs that target orexin/hypocretin receptors, and off-label use of medications such as other antidepressants, antihistamines, herbal preparations, and antipsychotics (50–52). However, these medications, including benzodiazepines and other hypnotics, might not be appropriate for treating sleep disturbance in AUD due to their potential for addiction and side effects, which can include residual sedation, memory and performance impairment, unwanted behaviors while sleeping, somatic symptoms, and drug interactions (53). Over more conventional types of treatment for sleep disorders, mindfulness-based therapies may offer a number of significant benefits: (1) mindfulness-based interventions can be given in a group setting over a short-term period, making them cost-effective; (2) MBSR is generally free of unwanted side effects and can be applied across a wide range of populations and disorders; and (3) attending a mindfulness class rather than engaging in other types of mental health therapy seems to be perceived by many individuals as having fewer stigmas. Thus, taken together, the results of the present study, MBSR may serve as an alternative treatment to hypnotics for sleep disturbance in patients with AUD.

There were several limitations to our study. First, the current study is a single-center study with a small sample size. Second, since the vast majority of Chinese patients with AUD are male patients, our hospital has only set up male inpatient areas. Thus, only male patients with AUD were included in the present study. The inability to examine gender differences may be considered a limitation. Third, the long-term effect of MBSR on the sleep quality of alcohol-dependent patients after withdrawal was not observed. Fourth, we cannot rule out the effect of the participants' mere time spent with experts on the results. It may be appropriate to add the same participants' mere time spent with experts in the control group to eliminate the influence of this unspecific factor.

At the end of this study, we concluded that short-term MBSR could improve sleep quality and may serve as an alternative treatment to hypnotics for sleep disturbance in patients with AUD after withdrawal. The exact mechanism and long-term effects of MBSR need further study to explore.

## Data availability statement

The original contributions presented in the study are included in the article/Supplementary material, further inquiries can be directed to the corresponding authors.

## Ethics statement

The studies involving human participants were reviewed and approved by the Ethics Committee of Hefei Fourth People's Hospital, Anhui Mental Health Center. The patients/participants provided their written informed consent to participate in this study.

## Author contributions

YW, CC, LP, and LX designed the study and wrote the protocol and the first draft of the manuscript. YW, CC, LG, YZ, YS, and GG performed the experiments. YX managed the literature searches and the statistical analyses. LP and LX edited the manuscript. All authors contributed to and have approved the final manuscript.
