# Supplementary material for: Effect of short-term mindfulness-based stress reduction on sleep quality in male patients with alcohol use disorder
Source: Front Psychiatry. 2023 Mar 14;14:928940. doi: 10.3389/fpsyt.2023.928940 (PMC10043304; doi:10.3389/fpsyt.2023.928940)
Supplement: Supplementary file 1 [file Table_1.DOCX]

**Table S1 Contents of MBSR training**

| 1. Meditative state of concentration (10 minutes) | The patients were placed in a sound insulation room with a constant temperature of 25 °C and asked to sit upright with their backs against the backrest of an armless chair, with the eyes naturally closed and the whole body relaxed. Then, the trained nurses used simple instructions to lead the patients to maintain smooth breathing. |
| --- | --- |
| 2. Meditation relaxation training (35 minutes) |  |
| 2.1 Breathing training (5 minutes) | The patient was guided to concentrate and adjust breathing to make it deep and long. |
| 2.2 Relaxation training (10 minutes) | The patient was guided to completely relax the muscles of the whole body from head to feet. |
| 2.3 Mind change awareness training (10 minutes) | The patient was guided to feel any subtle changes in mind, and experience the changes of the body from feet to head. |
| 2.4 Mindfulness meditation training (10 minutes) | The patient was guided to imagine pleasant things or scenes to completely relax body and mind. |
| 3. Meditation ending | The patient was guided to slowly open eyes and end meditation. |
